# An unedited 1.1 kb mitochondrial *orfB *gene transcript in the Wild Abortive Cytoplasmic Male Sterility (WA-CMS) system of *Oryza sativa *L. subsp. *indica*

**DOI:** 10.1186/1471-2229-10-39

**Published:** 2010-03-02

**Authors:** Srirupa Das, Supriya Sen, Anirban Chakraborty, Papia Chakraborti, Mrinal K Maiti, Asitava Basu, Debabrata Basu, Soumitra K Sen

**Affiliations:** 1 Advanced Laboratory for Plant Genetic Engineering (formerly IIT-BREF Biotek), Indian Institute of Technology, Kharagpur- 721302, India; 2 Dept of Pathology, Baylor College of Medicine, One Baylor Plaza, S209 Houston, Texas 77030, USA; 3 Stein Clinical Res Bldg 201, California University, San Diego, La Jolla CA 92093-0673, USA; 4 Bramhanand KC College, Kolkata- 700 035, India; 5 Bose Institute, Kolkata- 700 009, India

## Abstract

**Background:**

The application of hybrid rice technology has significantly increased global rice production during the last three decades. Approximately 90% of the commercially cultivated rice hybrids have been derived through three-line breeding involving the use of WA-CMS lines. It is believed that during the 21^st ^century, hybrid rice technology will make significant contributions to ensure global food security. This study examined the poorly understood molecular basis of the WA-CMS system in rice.

**Results:**

RFLPs were detected for *atp6 *and *orfB *genes in sterile and fertile rice lines, with one copy of each in the mt-genome. The RNA profile was identical in both lines for *atp6*, but an additional longer *orfB *transcript was identified in sterile lines. 5' RACE analysis of the long *orfB *transcript revealed it was 370 bp longer than the normal transcript, with no indication it was chimeric when compared to the genomic DNA sequence. cDNA clones of the longer *orfB *transcript in sterile lines were sequenced and the transcript was determined unedited. Sterile lines were crossed with the restorer and maintainer lines, and fertile and sterile F_1 _hybrids were respectively generated. Both hybrids contained two types of *orfB *transcripts. However, the long transcript underwent editing in the fertile F_1 _hybrids and remained unedited in the sterile lines. Additionally, the editing of the 1.1 kb *orfB *transcript co-segregated with fertility restoring alleles in a segregating population of F_2 _progeny; and the presence of unedited long *orfB *transcripts was detected in the sterile plants from the F_2 _segregating population.

**Conclusion:**

This study helped to assign plausible operative factors responsible for male-sterility in the WA cytoplasm of rice. A new point of departure to dissect the mechanisms governing the CMS-WA system in rice has been identified, which can be applied to further harness the opportunities afforded by hybrid vigor in rice.

## Background

The development of hybrid crops with improved yield characteristics is vital to meet the food needs of an increasing world population, assure sustainable land practices and contribute to ongoing conservation efforts. Hybrid rice has enabled China to reduce the total land used for planting from 36.5 Mha in 1975 to 30.5 Mha in 2000, while increasing production from 128 to 189 million tons [1]. Production of hybrid seeds in self-pollinating crop species requires the use of male-sterile plants. Cytoplasmic male sterility (CMS) is most commonly employed in developing such hybrids. CMS is a maternally-inherited trait that leads to failure in the production of viable pollen. [2] suggested it is the result of incompatible nuclear and mitochondrial functional interactions. Despite the existence of a number of different types of CMS systems, two key features are shared: (i) CMS is associated with the expression of chimeric mitochondrial open reading frames (ORFs); and (ii) fertility restoration is often associated with genes thought to regulate the expression of genes encoded by organellar genomes; for example, pentatricopeptide repeat (PPR) proteins involved in processing organellar RNAs [3,4]. In many cases, including rice, nuclear-encoded fertility restorer (*Rf*) gene(s) can restore male fertility. Consequently, sterility results from mitochondrial genes causing cytoplasmic dysfunction and fertility restoration relies on nuclear genes that suppress cytoplasmic dysfunction.

In almost all plant CMS systems studied to date, the male sterility trait was associated with changes in mitochondrial gene organization. [4] demonstrated that cytoplasmic male sterility was caused by protein defects involved in mitochondrial energy production and often involved ATP synthase subunit genes. Therefore, impaired ATP synthase activity could be a causal factor in disrupted pollen function. In several cases, mt-DNA rearrangement has been shown to generate novel chimeric ORFs, which resulted in the expression of novel polypeptides [5]. Often, these chimeric ORFs were adjacent to normal mitochondrial genes and sometimes the rearrangements resulted in the deletion of genuine mitochondrial genes [5,6]. To date, more than 50 genes associated with CMS have been identified in the mitochondria of a variety of plant species [7-10]. The sequences that contribute to the generation of the chimeric ORFs are typically derived from coding and noncoding regions of existing genes, but are occasionally from unknown origins. In most cases, impairment of functions of mitochondrial genes have been shown to be associated with CMS [4,5,11,12]. However, the precise relationship between mitochondrial CMS-associated genes and male sterility varies from species to species and is poorly understood.

A unique feature of plant mitochondrial gene expression is RNA editing, first detected by [13]. Generally, changes in the primary transcript involve C to U transitions by cytosine deamination. The editing process can change the amino acids that are encoded by mRNA, and also introduce new start and stop codons. Editing is essential to generate operative gene products (i.e. proteins). The functional relevance of plant mitochondrial RNA editing is high, as it results in the production of conserved polypeptides. In the presence of RNA editing, in some cases mature proteins are quite different in size, amino acid composition and function from that predicted in the genomic DNA sequence [14].

Commercially cultivated hybrid rice includes three-line and two-line hybrid rice developed through cytoplasmic male-sterility and photo/thermo-sensitive male sterility (PGMS/TGMS) [15], respectively. Furthermore, various types of CMS systems have been identified in rice, i.e., CMS-WA, CMS-HL and CMS-BT. Currently, the CMS-WA (wild abortive) system derived from the wild species *Oryza rufipogon *Griff [16] is applied most often for hybrid rice production [17]. Rice breeders tend to employ the CMS-WA preferentially as it gives stable CMS lines, restorers are frequently found and there is no indication of its genetic vulnerability to disease. However, the uniformity of the WA cytoplasm can result in genetic vulnerability to disease and insect pests. To overcome this, it is essential that the genetic source of CMS be diversified. Additionally, CMS requires the development and maintenance of separate male and female (seed) gene pools. Generally, only a subset of the female genotypes contains the genetic information required to reliably confer the desired phenotype. The female gene pools are often less diverse than the male gene pools, therefore the genetic diversity of the hybrid cultivars depends largely on variation in the male genotypes. This has been a major constraint for plant breeders. Thus, understanding the molecular basis of CMS in rice WA-cytoplasm is critical if improvements in rice hybrid seed production technology are to continue. The present study served to elucidate the molecular mechanisms conferring cytoplasmic male sterility in the WA system of CMS rice. Our initial investigation in the CMS-WA system evaluated the structural organization of certain mitochondrial genes that were previously implicated in CMS in various plant species, including *atpA*, *atp9*, *atp6 *and *orfB*. Here we provide experimental evidence for polymorphisms in *atp6 *and *orfB *structural organization and mitochondrial transcript profiles of the *orfB *gene in the CMS-WA rice system. The sterile line *orfB *gene transcript profile was characterized by two transcripts of ~1.1 kb and ~0.7 kb, and one ~0.7 kb transcript was detected in the fertile lines. The ~1.1 kb transcript present in the sterile line remained unedited. However, in the presence of nuclear encoded restoration of fertility (*Rf*) gene(s) in fertile restored hybrid lines (*APMS-6A *× *BR-1870*; F_1 _generation), the ~1.1 kb *orfB *transcripts were fully edited. The editing of the *orfB *gene ~1.1 kb transcript co-segregated with fertility restoring alleles in a segregating population of F_2 _progeny of restored hybrid F_1 _plants.

## Results

### Structural organization of *atp6*, *atpA*, *atp9 *and *orfB *in sterile and fertile rice lines

**Figure 1:**
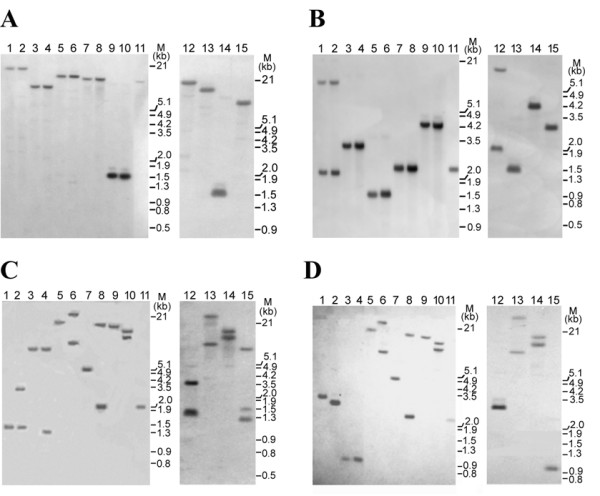
**RFLP analysis of sterile, maintainer and restorer rice lines for *atp9*, *atpa *and *atp6 *genes**. Southern blot analysis of the *APMS-6A *WA sterile line (lanes: 1, 3, 5, 7, 9) along with the corresponding maintainer *APMS-6B *(lanes: 11, 12, 13, 14, 15) and restorer *BR-1870 *(lanes: 2, 4, 6, 8, 10) lines. Mitochondrial genomic DNA (10 μg per lane) was digested with different restriction enzymes, viz., *Bgl*II (lanes: 1, 2, 12), *Sca*I (lanes 3, 4, 15), *Dra*I (lanes 5, 6, 13), *Eco*RI (lanes 7, 8, 11) &*Hin*dIII (lanes 9, 10, 14), run on an 0.8% agarose gel, blotted and probed with different mitochondrially-encoded CDSs or partial CDSs. Lane M: *Eco*RI and *Hin*dIII-digested phage λ DNA (molecular weight marker). **Panel A: **Southern blots probed with the entire CDS of the *atp9 *gene. **Panel B: **The same blots were stripped and re-probed with the partial CDS of the *atpA *gene. **Panel C: **The same blots were re-probed with the partial CDS of the *atp*6 gene. **Panel D: **The same blots were re-probed with the 3'UTR of the *atp*6 gene.

**Figure 2:**
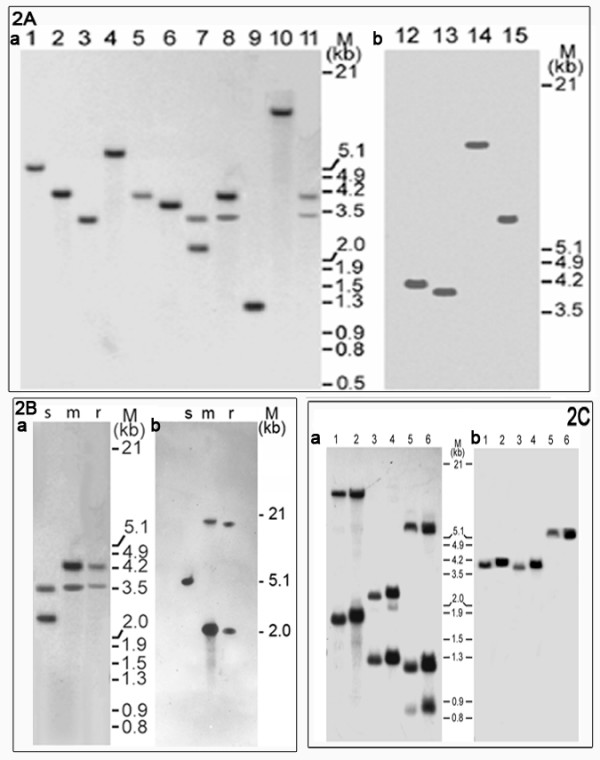
**RFLP analysis of sterile, maintainer and restorer rice lines for *orfB *and *atp6 *genes**. **2A**. Southern blot analysis of the *APMS-6A *WA sterile line (lanes: 1, 3, 5, 7, 9) along with corresponding maintainer *APMS-6B *(lanes: 11, 12, 13, 14, 15) and restorer *BR-1870 *(lanes: 2, 4, 6, 8, 10) lines. The same blots that were shown in Figure 1 were stripped and re-probed with the CDS of the *orfB *gene. **2B**. DNA Gel Blot Analysis of WA-CMS line *IR58025A*(s), *IR58025B*(m) and its *restorer*(r). a. Mitochondrial DNA digested with *Eco*RI restriction enzyme and probed with rice *orfB *CDS. b. Same blot stripped and probed with *atp6 *partial CDS. **2C**. DNA Gel Blot Analysis of non WA-CMS rice line, *Kalinga-32A *and corresponding fertile maintainer line, *Kalinga-32B Kalinga-32A *(lane 1, 3, & 5) and *Kalinga-32B *(lane 2, 4, & 6) mitochondrial DNA (10 μg) digested with three different restriction enzymes, viz., *Eco*RI (lanes 1 & 2), *Bgl*II (lanes 3 & 4) and *Sca*I (lane 5 & 6), were electrophoresed, blotted and probed with rice *atp6 *CDS. Same blot probed with *orfB *CDS.

The organization of four mitochondrion-encoded genes was examined by Southern blot analysis of the CMS rice line *APMS-6A*, including the corresponding maintainer *APMS-6B *and restorer *BR-1870 *lines. The analysis was conducted with mitochondrial genomic DNA. However, it was determined that analysis of total cellular DNA of each experimental line revealed the same restriction fragment length polymorphism (RFLP) pattern as mitochondrial DNA with respect to the mt-genes under consideration. Restriction fragment length polymorphisms were not observed for *atp9 *or *atpA *in any of the three rice lines *APMS-6A*, *APMS-6B*, and *BR-1870 *(Figures 1A and 1B). The *atp9 *probe hybridized to a single restriction fragment (Figure 1A) with all five restriction enzymes, indicating the existence of a single copy of the gene. The *atpA *gene exhibited the same results, with the exception of *Bgl*II, where the *atpA *probe detected a 2.1 kb and 12 kb fragment in all three lines (Figure 1B) due to the presence of a *Bgl*II site within the 720 bp probe sequence. However, RFLPs were detected in the *atp6 *gene between the *APMS-6A, APMS-6B *and *BR-1870 *lines (Figures 1C and 1D). The sterile lines contained a single band, whereas the fertile maintainer and restorer lines showed two hybridizing bands each for all five restriction enzymes. *Sca*I exhibited an additional 1.6 kb fragment hybridized to the partial *atp6 *coding region probe in the maintainer rice line. A polymorphism was also evident when the *atp6 *3'-untranslated region (UTR) was used as a probe (Figure 1D). Additionally, RFLPs were observed for the *orfB *gene (Figure 2A) in the mitochondrial genome between the sterile and the fertile lines. All restriction enzymes with the exception of *Eco*RI gave rise to a single hybridizing band with size variation between the sterile and fertile lines. Due to the presence of an *Eco*RI site in the *orfB *gene probe, digestion with *Eco*RI consistently generated two bands in all rice lines. The length of one band varied between the sterile and the fertile lines. Therefore, it was evident that mitochondrial *orfB *gene was present as a single copy with differential organization in the sterile and fertile lines. This was based on observations that with the exception of *Eco*RI, all restriction enzymes gave rise to single hybridizing bands of variable sizes in fertile and sterile rice lines. The results of the Southern blot analysis are represented in supplementary (Additional file 1 and 2). Additionally, RFLPs were also tested for mitochondrial *atp*6 and *orf*B genes in *Eco*RI digested mitochondrial DNA of CMS-WA *IR58025A *(sterile), *IR58025B *(maintainer) and the restorer (*BR-1870*) lines (Figure 2B). The band patterns were exactly similar to observations made in case of the *APMS6A/B *and restorer lines. Furthermore, the mitochondrial DNA of a non WA-CMS system in rice, *Kalinga 32A/B *lines, was also tested for RFLP studies with *atp*6 and *orf*B genes. In this case, no DNA band polymorphism was observed (Figure 2C).

### Transcription profile of polymorphic *atp6 *and *orfB *genes

**Figure 3:**
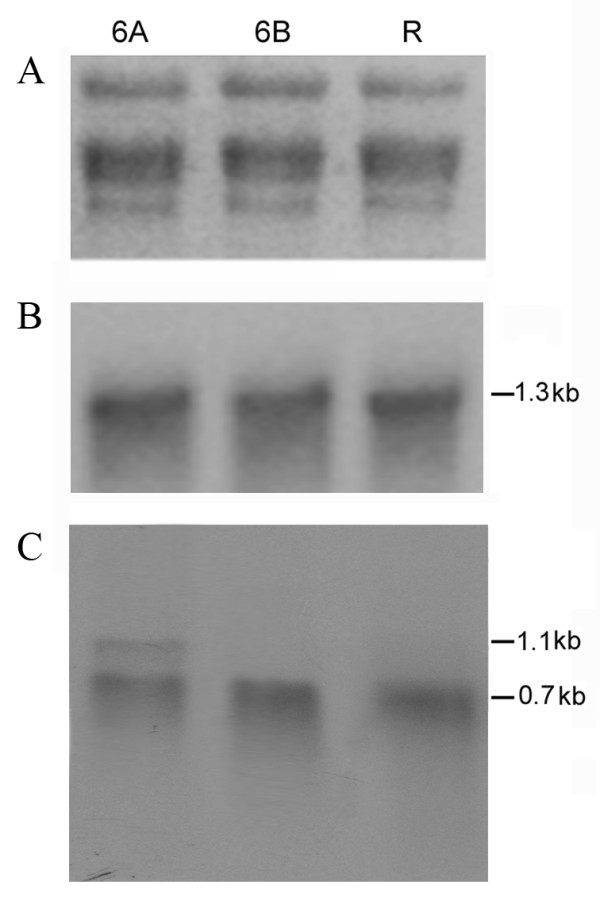
**Northern blot analysis of WA sterile, maintainer and restorer rice lines for the presence of *atp6 *and *orfB *transcripts**. Approximately 10 μg of total mitochondrial RNA from the leaves of sterile (6A), maintainer (6B) and restorer (R) lines were loaded on a 1.2% denaturing formaldehyde gel. (A) Equal loading of RNA samples from the three lines was shown by visualization of the ribosomal RNA bands by staining the gel in ethidium bromide before blotting. (B) Autoradiograph of the blot hybridized with the *atp*6 gene-specific probe. (C) Autoradiograph of the same blot after stripping and reprobing with the rice *orfB *gene-specific probe.

Mitochondrial RNA Northern blot analysis from sterile, maintainer and restorer rice lines was performed to determine if DNA polymorphisms in the *atp6 *and *orfB *gene loci resulted in changes in expression profiles for these two genes (Figure 3). Radiolabelled probes for the respective genes were generated for carrying out the evaluation. A single ~1.3 kb transcript was detected for the *atp6 *gene in both sterile and fertile lines (Figure 3, Panel B). Thus, the *atp6 *gene expression was not influenced due to the DNA polymorphism as observed between the *atp6 *loci in sterile and fertile mitochondria. In contrast, differences in *orfB *gene transcripts were observed between the WA sterile and fertile maintainer and restorer lines. The *orfB *probe detected a single ~0.7 kb transcript in the male-fertile maintainer and restorer lines, whereas in the WA sterile line, a transcript of ~1.1 kb with a relatively lower intensity was observed in addition to the major ~0.7 kb *orfB *transcript (Figure 3, Panel C). Northern blot analysis with strand-specific probes confirmed that all transcripts from each genotype were of the same polarity (data not shown).

### Editing of the *orfB *transcripts

#### (a) The fertile line

**Figure 4:**
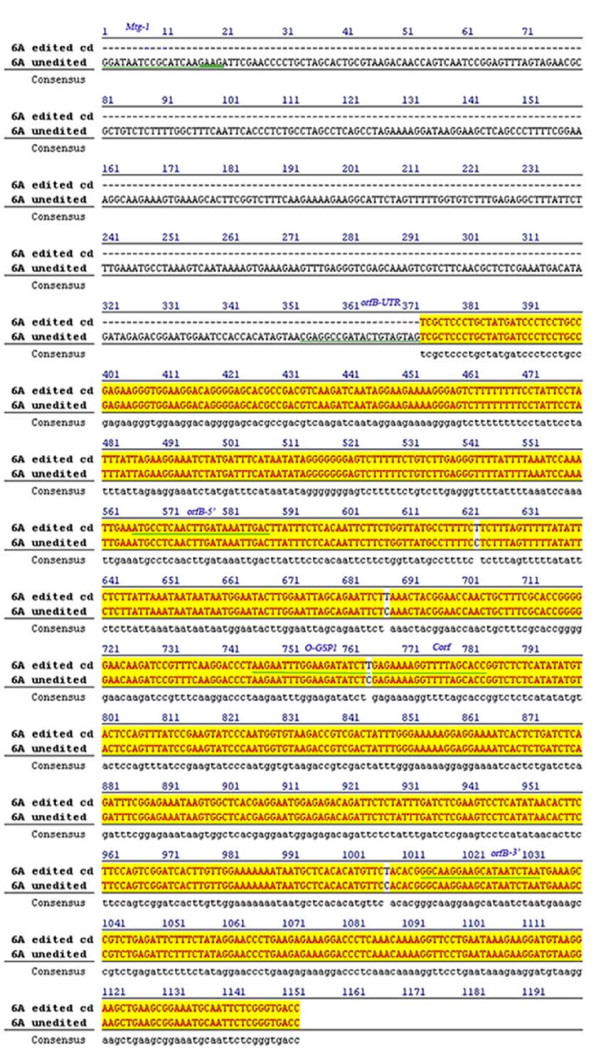
**Sequence alignment of 0.7 kb and 1.1 kb transcripts of *orfB *gene**. Position of primers used in RT-PCR and RACE experiments are shown in the sequence alignment of the edited ~0.7 kb and unedited ~1.1 kb transcripts of the *orfB *gene. The CDS is from 566-1033. The alignment was performed with Jellyfish version 1.3 software provided by biowire.com.

Mitochondrial RNA editing of the *orf*B transcript was assessed in the fertile rice line. Fourteen cDNA clones obtained from cDNA library of fertile rice line were sequenced. Determination of the *orfB *cDNA sequence from overlapping clones from the cDNA library showed four C→T conversions within the coding region relative to the *orfB *genomic sequence. Two editing events within the coding region affected the second position in a codon (200^th ^and 443^rd^), and another event changed the first position (58^th^). These three editing events altered the coding properties of the affected triplets, which led to major changes in amino acids [Leu→Phe (20^th^), Ser→Leu (67^th^) and Pro→Leu (148^th^)]. Furthermore, editing at nucleotide position 200 in the coding region of *orfB *disrupted an *Xho*I restriction site (C**TCG**AG to C**TTG**AG). The fourth substitution was at the third position of a codon for leucine and was silent (Figure 4). Results showed that all four sites within the coding region were edited in all 14 clones. This indicated highly efficient and consistent mitochondrial editing for this transcript in the fertile rice line.

#### (b) The sterile line

*orfB *cDNA sequences were determined from overlapping clones of the cDNA library from the sterile rice line. Twelve *orf*B cDNA clones were completely sequenced. The size of the inserts ranged from 647 bp to 230 bp. Analysis of the clones revealed that they comprised sequences that overlapped with each other and were homologous to the nucleotide sequence of *orfB *cDNA from the fertile line (Figure 4). However, in contrast to the cDNA clones from the fertile line, unedited as well as edited cDNA clones were obtained from the sterile line. The edited clones exhibited identical editing to the cDNA clones in the fertile line. Interestingly, however, in the clone with the largest insert (6A25-11) editing was absent. Sequence analysis also indicated the insert contained a portion of the 5' UTR region of the *orf*B gene, not detected in 0.7 kb *orf*B gene transcripts of the fertile lines. It was therefore inferred that the clone contained an insert originating from the long 1.1 kb transcript of the *orfB *gene. Furthermore, an additional interesting clone (6A21-61) of 230 bp was detected. It contained three unedited sites; unlike the other two clones that contained one unedited site out of four, normally found edited within the *orfB *gene coding sequence (CDS). Observing that some of the *orf*B gene transcripts in the sterile line remain unedited appeared significant.

### *orfB *transcripts of the sterile line have identical 3' ends with that of transcripts from the fertile lines

**Figure 5:**
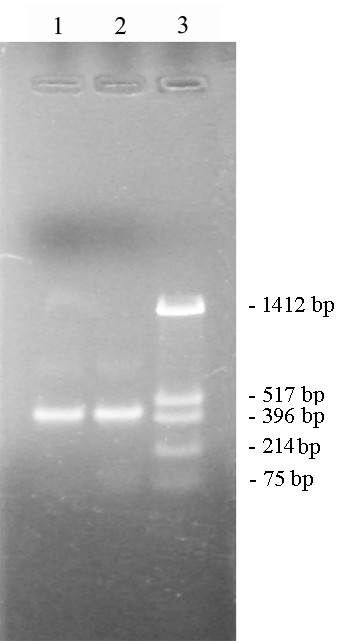
**3'- RACE of *orfB *gene transcripts**. 3'- RACE PCR product run on a 1% agarose gel. Lane 1: 3'- RACE product from the sterile line. Lane 2: 3'-RACE product from the fertile line. Lane 3: Molecular marker (pUC18/*Hin*fI).

The basis of the observed differences in the *orfB *gene transcripts between the sterile and fertile lines was determined using 3' RACE. The forward primer O-GSP1 (Figure 4) annealed 180 bp downstream of the initiation codon in the coding region of the *orfB *gene. In both the fertile and sterile rice lines, one amplified band of ~400 bp was obtained (Figure 5). All the amplified products from the sterile and fertile lines were cloned into the pUC18 vector. More than 20 clones were randomly selected and sequenced. It was confirmed by hybridization with the *orfB *CDS gene probe that all clones contained the desired insert (data not shown). Fertile line sequencing revealed all clones were edited, whereas in the sterile line, both edited and unedited clones were observed. All clones from fertile and sterile lines contained a 120 bp 3' UTR in addition to the partial CDS region. Thus, the edited and unedited *orfB *transcripts from the sterile and fertile genotypes were 3' co-terminal and terminated 120 bp downstream of the translation termination codon TAA.

### *orfB *transcripts have differential 5' UTR regions in fertile and sterile lines

**Figure 6:**
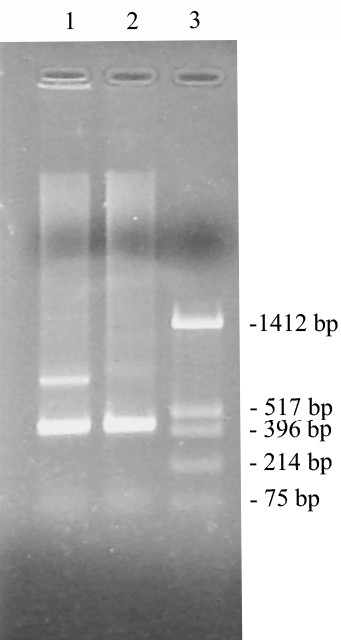
**5'- RACE of *orfB *gene transcripts**. 5'-RACE PCR product from the sterile and fertile rice lines run on a 1.0% agarose gel. Lane 1: 5'-RACE product of the sterile rice line. Lane 2: 5'-RACE product of the fertile rice line. Lane 3: Molecular weight marker (pUC18/*Hin*fI).

Characterization of the *orfB *transcript 5' upstream region of the sterile and fertile rice lines was performed by mitochondrial cDNA 5' RACE using the Corf primer. The primer annealed 201 bp downstream of the initiation codon. Two bands of approximately ~750 bp and ~400 bp were generated in the sterile *APMS-6A *rice line (Figure 6, lane 1). One ~400 bp product was observed in the fertile *BR-1870 *rice line (Figure 6, lane 2). PCR products were individually cloned into pUC18. Positive clones were identified for sequencing by hybridization with the *orfB *CDS probe. Random sequencing of 18 clones of ~750 bp PCR products from the sterile rice line revealed a 5' UTR of 565 bp in addition to the 201 bp partial CDS. Sequencing of 16 clones of ~400 bp 5' RACE product revealed a 5' UTR of 192 bp in addition to the 201 bp partial CDS. The clones with the longer 5' UTR were unedited, as was evident from the sequence of the 201 bp fragment of the coding region, where as the clones with the shorter 5' UTR were edited. In case of the fertile rice line, sequencing of 18 clones obtained with the ~400 bp 5' RACE product revealed *orfB *transcripts with a 5' UTR of 192 bp only. They were completely edited.

Sequence analysis showed that, despite the larger size of the unedited transcript, the coding region was identical to that of the smaller edited transcript, with the exception of four single nucleotide changes that arose from editing. The 565 bp 5' UTR sequence of the ~1.1 kb transcript was identical to the rice mitochondrial genomic sequence (Acc# DQ167399). The entire edited ~0.7 kb and unedited ~1.1 kb *orfB *gene transcript sequences are shown in Figure 4.

**Figure 7:**
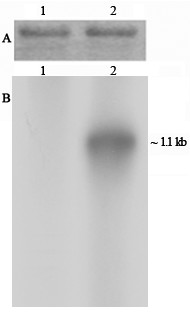
**Northern blot analysis of sterile and restorer rice lines in search of 1.1 kb transcript**. Northern blot analysis of the WA sterile (lane 2) and restorer (lane 1) rice lines using the PCR product obtained by the primer set Mtg-1 and orfB-UTR as probe. (A) Equal loading of RNA samples was shown by visualization of ribosomal RNA bands by staining the gel in ethidium bromide before blotting. (B) Autoradiograph of the blot after probing with ~1.1 kb transcript specific probe.

Following assembly of the partial sequences obtained from the cDNA library, 3' RACE and 5' RACE experiments, the entire ~1.1 kb and ~0.7 kb transcript sequences were deciphered. To test the accuracy of the ~1.1 kb specific sequence, a Northern blot analysis was performed with mitochondrial RNA from sterile and fertile restorer rice lines (Figure 7). The 5' genomic DNA upstream of the ~0.7 kb transcript sequence was chosen as the radiolabelled probe. The fragment was PCR amplified using the primer set Mtg-1 and orfB-UTR (Figure 4). A ~1.1 kb fragment was detected in the sterile line but not in the restorer rice line (Figure 7, panel B). It should be noted that in the sterile line, Northern blot analysis using *orfB *CDS as the probe generated both ~0.7 kb and ~1.1 kb bands; while the fertile restorer rice line revealed only the ~0.7 kb transcript. Therefore, this result confirmed the extensive 5' UTR belonged to the ~1.1 kb transcript.

### RT-PCR analysis reveals that the ~1.1 kb transcript does not undergo editing in sterile rice lines

**Figure 8:**
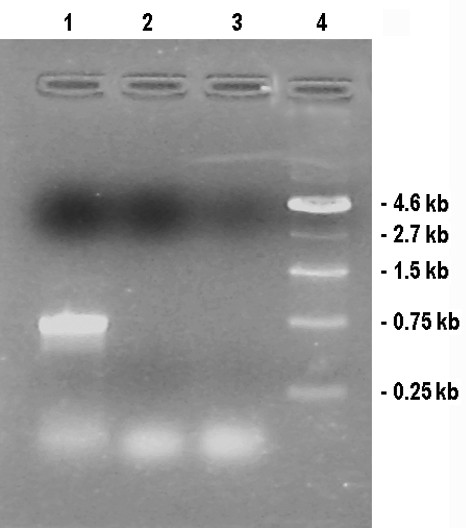
***OrfB *gene 1.1 kb transcript specific RT-PCR from sterile rice line**. Ethidium bromide stained agarose gel (1%) showing the RT-PCR product using gene specific Mtg-1 and Corf primers from WA-sterile rice line (lane 1). Lane 2 and lane 3 show the absence of the band in the maintainer and the restorer rice lines, respectively. Lane 4: Molecular weight marker.

The RNA editing status of the ~1.1 kb transcript was evaluated in the sterile rice line (*APMS-6A*). RT-PCR analysis was performed using the 5' gene specific primer Mtg-1 (which annealed at the far end of the 5' UTR region of the ~1.1 kb transcript) and 3' gene specific primer Corf (which annealed 201 bp down stream of ATG) (Figure 4). The Mtg-1 primer annealed only to the longer ~1.1 kb transcript. RT-PCR generated a band of ~770 bp (Figure 8, lane 1); maintainer and restorer rice lines do not possess the ~1.1 kb transcript; consequently amplification was absent (Figure 8, lanes 2 and 3). Twenty randomly selected clones from this RT-PCR product were sequenced and revealed the presence of only unedited clones. Sequencing could aid in detection, as three editing sites fell within the partial CDS region chosen for RT-PCR amplification. It was therefore evident that ~1.1 kb transcript remained essentially unedited in the WA-sterile rice line.

### Transcript profile of the *orfB *gene in maintained hybrid (*APMS-6A *× *APMS-6B*) and restored hybrid (*APMS-6A *× *BR-1870*) lines

**Figure 9:**
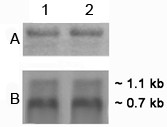
**Northern blot analysis of *6AB *and *6AR *F_1 _plants**. Northern Blot Analysis of the progeny of *APMS-6A *× *APMS-6B *(lane 1) and *APMS-6A *× *BR-1870 *(lane 2) crosses using the *orfB *gene probe. (A) Equal loading of RNA samples was shown by visualization of the ribosomal RNA band visualized by staining the gel in ethidium bromide before blotting. (B) Autoradiograph of the blot after probing with the rice *orfB *gene-specific probe.

**Figure 10:**
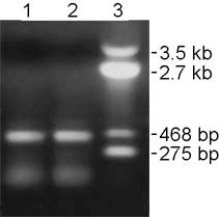
**RT-PCR of *orfB *CDS from *6AB *and *6AR *F_1 _plants**. Ethidium bromide-stained agarose gel (1%) showing the PCR-amplified products of the complete *orfB *CDS from the cross of *APMS-6A *with the maintainer line *APMS-6B *(lane 1) and the restorer line *BR-1870 *(lane 2). Lane 3: DNA molecular weight marker.

The influence of the nuclear encoded *Rf *alleles on transcription of the *orfB *gene was tested in two types of F_1 _plants, sexual hybrids *APMS-6A *× *APMS-6B *(maintainer) and *APMS-6A *× *BR-1870 *(restorer). Pollen produced by the restored F_1 _(sterile × restorer) plants was viable. However, pollen produced by the F_1 _(sterile × maintainer) plants was sterile. Northern blot analysis of mt-RNA of both types of F_1 _plants was carried out with the radiolabelled CDS region of the *orfB *gene as the probe. Northern blot analysis (Figure 9, panel B) revealed the presence of two bands, a ~0.7 kb and a longer ~1.1 kb band in the maintainer and restorer F_1 _plants. Subsequently, the *orfB *gene coding region was isolated from both hybrid lines by RT-PCR. Amplification with the orfB-5' and orfB-3' gene-specific primers produced a 468 bp product for both hybrid lines (Figure 10). Thirty-two clones of the maintainer F_1_(*APMS-6A *× *APMS-6B*) plants were randomly selected and sequenced and provided evidence for the presence of edited and unedited sequences. About 71.87% of the clones were edited, while the remaining 28.13% were unedited. However, sequence analysis of an equal number of clones in restorer F_1 _(*APMS-6A *× *BR-1870*) plants revealed the presence of only edited sequences. This indicated the ~1.1 kb *orfB *transcripts experienced editing under the influence of the *Rf *gene present in the restorer line.

### The longer ~1.1 kb *orfB *transcript of WA-cytoplasm remains unedited in the absence of nuclear encoded restoration of fertility (*Rf*) alleles

**Figure 11:**
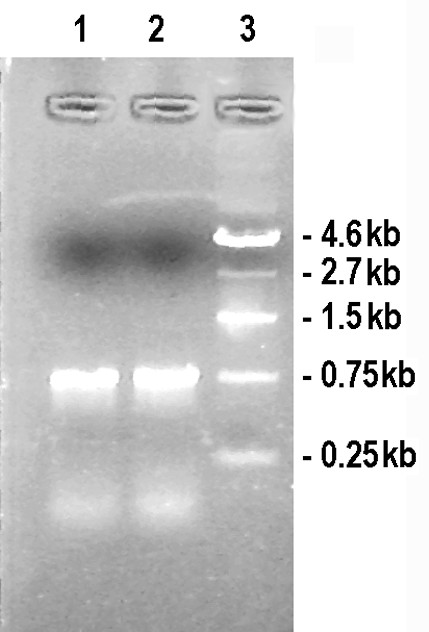
***orfB *gene 1.1 kb transcript specific RT-PCR from *6AB *and *6AR *F_1 _plants**. Ethidium bromide stained agarose gel (1%) showing the RT-PCR products using gene specific primers Mtg-1 and Corf from the cross of *APMS-6A *with the maintainer line *APMS-6B *(lane 1) and the restorer line *BR-1870 *(lane 2). Lane 3: DNA molecular weight marker.

In order to test the influence of the nuclear encoded fertility (*Rf*) restorer alleles on the editing of the ~1.1 kb *orfB *gene transcript, a separate RT-PCR experiment was conducted. The maintainer F_1 _sterile plants and the fertility restorer F_1 _plants were subjected to RT-PCR analysis using the 5' gene specific Mtg-1 and the 3' gene specific Corf primers (Figure 11). The ~770 bp RT-PCR products were cloned and for maintainer and restorer plants, 15 randomly selected clones were sequenced. The results showed the presence of only unedited clones in the maintainer sterile lines but the restorer hybrids exhibited edited clones.

### The edited phenotype of ~1.1 kb *orfB *transcript co-segregates with the restoration of fertility (*Rf*) alleles

**Figure 12:**
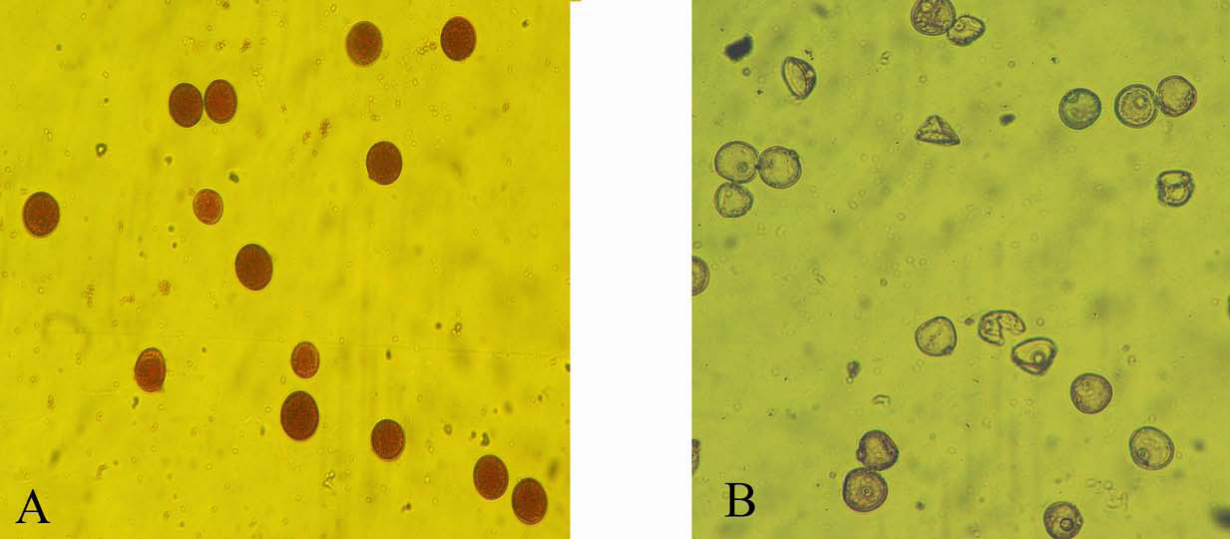
**Test for pollen fertility**. Representative microscopic view (40× magnification) of aceto-carmine stained pollen from F_2 _generation hybrid plants (*APMS-6A *× *BR-1870*). (A) Fertile plant (B) Sterile plant.

**Figure 13:**
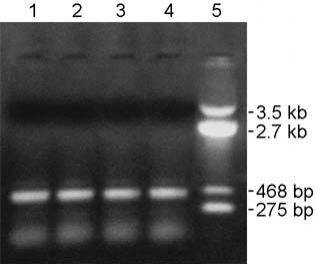
**RT-PCR of *orfB *CDS from *6AR *F2 sterile and fertile plants**. Ethidium bromide stained agarose gel (1%) showing the RT-PCR amplified products of the *orfB *CDS from the F_2 _progeny of the cross *APMS-6A *with the restorer line *BR-1870*. Lane 1 and lane 2 from sterile plants. Lane 3 and lane 4 from fertile plants. Lane 5: DNA molecular weight marker.

**Figure 14:**
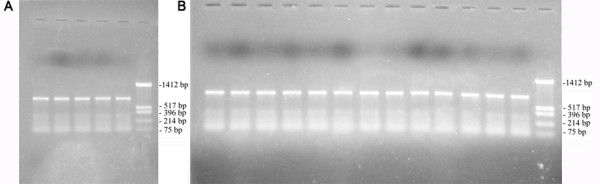
***OrfB *gene 1.1 kb transcript specific RT-PCR from *6AR *F_2 _sterile and fertile plants**. Ethidium bromide stained agarose gel (1%) showing the RT-PCR products using gene specific primers Mtg-1 and Corf from the F_2 _plants. The electropherograms show: (A) all the five F_2 _sterile plants identified; (B) 13 fertile plants as representative of 24 F_2 _fertile plants that were analyzed. DNA molecular weight marker used (pUC18/*Hinf*I).

One hundred sixty-two F_2 _progeny from the *APMS-6A *× *BR-1870 *cross were raised in the field in summer 2008. A screening for male-sterile plants on the basis of pollen fertility among the F_2 _progeny resulted in identification of two sterile segregant plants (Figure 12). The two sterile plants and the randomly selected two fertile plants among the 2008 F_2 _segregant progeny were subjected to RT-PCR analysis. The *orfB *gene coding region was investigated using orfB-5' and orfB-3' gene specific primers. In all F_2 _plants, 468 bp products were amplified via PCR (Figure 13). Both edited and unedited clones were found in the two sterile plants, whereas only edited clones were detected in the two fertile plants. Subsequently, in a repetitive experiment, 212 F_2 _progeny plants of the same cross combination were raised in summer 2009, to screen for sterile segregant plants. Three sterile plants were identified among the 212 plants. Thereafter, the 5 sterile plants (two and three from 2008 and 2009 F_2 _population, respectively) and 24 randomly selected fertile plants from F_2 _population of the 2009 season were subjected to RT-PCR analysis using the Mtg-1 and Corf primers. The ~770 bp RT-PCR products (Figure 14A and 14B) were cloned and randomly selected 14 clones from each plant line were sequenced. Only unedited clones were found in all the F_2 _sterile plants. In case of 24 fertile plants, on the contrary, only edited clones could be found in all cases of 336 clones analyzed. Thus, the results provided a case for the presence of a strong correlation between non-editing of the *orfB *~1.1 kb transcript and the sporophytic male sterility phenotype in the CMS-WA system.

### Inheritance pattern of restoration of fertility trait amongst F_2 _progenies

The F_2 _progenies of a cross made between *APMS-6A *× *BR-1870 *in 2007 were raised in 2008 and also in 2009 cropping season. A search was made to identify individual plants with sterile pollen amongst the segregating plant population for restoration of fertility genes/alleles. In 2008, there were 2 sterile plants out of 162 plants scored. In 2009, likewise, 3 plants with sterile pollen could be found amongst 212 plants. Based on the past information that the restoration of fertility nuclear genes (*Rf/rf*) are located in chromosome 1 and 10 of the rice genome, the segregation pattern of the restoration of fertility genes in the present case was studied. The observation of each season was subjected to chi-square test hoping for the *Rf *genes to assort independently into the gametes. In the present case, there were two phenotypic classes, viz., plants with fertile pollen and plants with sterile pollen. The frequency of appearance of sterile plants from this particular cross combination was too low to fit in dihybrid pattern of epistatic gene interaction (15:1). However, the observed number of segregation of two phenotypic classes fitted well with trihybrid cross ratio of 63:1 as per chi-square test, when epistatic gene interaction could be operative (see Additional file 3). The chi-square test result indicated that the experimental data provided no statistically compelling argument against this hypothesis.

## Discussion

Previous RFLP analyses in several plant species have provided evidence for mt-DNA genome organizational differences between cytoplasmic male-sterile and male-fertile lines. In some cases, these differences helped to identify genetic elements responsible for the CMS trait [18-22]. In the present study, similar analysis of rice mt-DNA with WA-CMS cytoplasm revealed variations associated with the *atp6 *(Figure 1C and 1D) and *orfB *(Figure 2A) loci in the sterile and fertile rice lines. The *orfB *gene, although present as single copy in sterile and fertile lines, exhibited polymorphisms in its structural organization. In addition, the *orfB *gene exhibited a differential transcript profile in the sterile lines relative to the fertile rice lines. Northern blot analysis revealed two, one ~0.7 kb and another ~1.1 kb sized *orfB *transcripts in the sterile lines; but only the ~0.7 kb transcript was detected in the fertile lines (Figure 3). The ~1.1 kb transcript in the sterile lines was characterized by a 565 bp 5'UTR, which is notably shorter in the ~0.7 kb transcript; both transcripts possess identical 120 bp 3' UTR regions. This indicates the larger transcript was likely transcribed from a different 5' initiation site located upstream of the gene. Alternatively, it could have arisen from an independent transcription initiation event. This would have been driven by an alternative promoter, located upstream of the promoter that normally drives expression due to a rearrangement in the mitochondrial genome. [23] reported the presence of multiple promoters driving expression of the maize *cox2 *gene. Promoter multiplicity has a marked influence on transcript complexity. However, an explanation for the generation of a longer *orfB *transcript in sterile lines is yet to be offered. [24] reported the presence of several structural variations between the WA-genome compared to fertile lines, and the mitochondrial expression profile between the genomes showed differential expression of only two mRNAs.

Interestingly however, the *orfB *gene CDS remains identical in both transcripts with the exception of four single nucleotide changes due to RNA editing. Furthermore, in WA-CMS the ~1.1 kb transcripts do not undergo editing. As a result, both edited and unedited *orfB *gene transcripts are formed in the sterile line. Alternatively, the fertile line is characterized by the presence of only edited transcripts. These changes (amino acid conversion due to RNA editing) could be functionally significant with respect to *orfB *gene function. Among these changes, nucleotide position 58, which corresponds to the 20^th ^amino acid, is highly conserved in plants and is located within the transmembrane helix of ORFB, as predicted by the SOSUI program (Mitaku Group, Department of Biotechnology, Tokyo University of Agriculture and Technology).

The *orfB *gene CDS in rice with WA cytoplasm was found to be identical to the CDS of *japonica *rice (Acc#. BA000029). The CDS from the WA cytoplasm rice line was also found to be homologous to the *atp8 *gene in other monocot species, for example, approximately 95% homologous to the *atp8 *gene in wheat (Acc#. AP008982); 96% homologous to the *atp8 *gene in sorghum (Acc#. DQ984518); and 94% homologous to the *atp8 *gene in corn (Acc#. DQ490953) (Additional file 4A). However, substantial divergence (Additional file 4B) between rice *atp8 *genes and dicot species has been reported, for example, 71.6% homology in *Beta vulgaris *(Acc#. NC002511); and 50.2% in *Daucus carota *(Acc#. AY007818). The *orfB *gene nucleotide sequence is also completely identical to the *orf156 *mitochondrial gene sequence of wheat [25]. The similarity of wheat *orf156 *to rice *orfB *gene transcripts extends further in that the editing takes place at the same four positions of the CDS, corresponding to three amino acid substitutions and one silent modification. Wheat *orf156 *encodes a polypeptide of 18 kDa that is associated with mitochondrial membrane function [25]. This is congruent with former suggestions that most CMS-associated mechanisms operative in plants follow a common comprehensive approach [5,11]. The observed transcript changes of *orfB *gene may not only be the result of the environment of the mitochondrial genome, but can also be affected by dominant nuclear genes [26]. *orfB *gene transcript profile analysis of F_1 _plants, derived from sexual crosses between CMS plants (*APMS-6A*) and isonuclear maintainer lines (*APMS-6B*), and between CMS plants (*APMS -6A*) and restorer lines (*BR-1870*) provided experimental evidence that ~1.1 kb transcripts of the *orfB *gene undergo editing under the influence of the nuclear encoded fertility restorer (*Rf*) alleles. The ~1.1 kb *orfB *transcript remained unedited in male-sterile lines. In maintainer and restorer hybrid plant lines, the two *orfB *transcripts were present; but the ~1.1 kb transcript was edited in the restorer hybrid lines. This is significant as we know in plants where the seed is harvested, it is imperative that the F_1 _hybrid be male fertile. Thus, the fundamental characteristics of CMS in rice have a bearing on this requirement. Differences in transcript profiles between CMS and fertile lines in the presence of fertility restorer genes have been observed in sunflower [27]. [28] reported that the *B-atp6 *gene transcript pattern, associated with CMS-BT rice carrying *cms-bo *cytoplasm, was altered in the presence of the nuclear *Rf-1 *gene. The *B-atp6 *gene was transcribed into a 2.0 kb RNA in the absence of the *Rf-1 *gene, but into two discontinuous RNAs (~1.5 kb and 0.45 kb) in the presence of the *Rf-1 *gene. In the present case, however, restoration of fertility does not lead to any change in the transcript profile of the *orfB *gene.

Despite the fact that some *Rf *loci are known to affect the transcript profile of CMS associated loci in several plant species, how the altered expression of *Rf *genes/alleles influence fertility restoration is not known [4]. In rice many pentatricopeptide repeat (PPR) gene allele are present, unlike wheat, maize and sorghum [29]. Many of these PPR genes are clustered in chromosomal regions [30,31], similar to radish and petunia. As the PPR clusters in rice genotypes are consistent with their chromosomal locations, it has been deduced that variations in the number of PPR gene/allele members and existence of null members in such clusters may exist in different genotypes of rice [31,32]. In such a situation, it is suspected that some of the members of PPR clusters function in collective manner as fertility restorers with distinct functional role to perform in a CMS system. Functional variations between a series of restoring *(Rf) *alleles and nonrestoring *(rf) *alleles are speculated to exist between genotypes including restorer lines in rice [30-32]. It was speculated in the past based on genetic data that the fertility of CMS-WA is controlled by one or two pairs of restorer alleles corresponding to different restorer lines [33,34]; and they function in an independent fashion in various restorer lines [35,36]. Based on this, it has generally been believed that in CMS-WA, two rice fertility restorer genes are required for the production of viable pollen. These genes have been mapped to chromosomes 1 and 10 [37-40]. However, segregation analysis of a F_2 _population for fertility restoration and genotyping using molecular markers revealed that fertility restoration in WA-system is controlled by more than two loci; one on the short arm of chromosome 1, one on the short arm of chromosome 10, one on the long arm of chromosome 10 and an unknown *Rf *gene [41]. The location of the *rf *gene remains unknown. Thus, it can be hypothesized that in the presence of different restoring (*Rf*) genes/alleles, differential epistatic influence on male sterility/fertility is operative. How PPR proteins regulate the restoration process remains, however elusive.

In most cases, fertility restoration is attained through nuclear-encoded *Rf*-gene-dependent mitochondrial RNA modification and concurrent reduction of the CMS-associated protein. The nature of the *Rf *genes that affect mitochondrial gene expression has long been considered a black box. Earlier studies have indicated the possible roles of restorer gene(s) on editing rice *atp6 *transcripts in CMS-BT [28,32] and sorghum A3 CMS [42]. However, the relationship between editing to fertility restoration remains unclear. Earlier authors [32] have shown that restorer genes increase the editing efficiency of *atp6 *transcripts in rice Bo-CMS cytoplasm. Based on the fact that the editing of the *orfB *transcripts in rice in the present case did not affect the reading frame, the unedited transcripts should hypothetically be translated and result in the production of a mutant form of the protein, as three of the codons that remained unedited alter the amino acids.

Reports indicate that most CMS-associated genes expressed at much higher levels in anther tissue than in seedlings [43,44] during micro-sporogenesis when ATP requirements are abnormally high [45]. High levels of F_0_F_1_-ATP synthase activity demonstrate that anther cells require more ATP than other tissues. Because cellular energy requirements are maximal in tapetal cells during microsporogenesis, reduced mitochondrial function in plants could result in pollen abortion. The present investigation detected an unedited ~1.1 kb *orfB *gene transcript in WA cytoplasm rice. It is plausible that competition between translated products of edited and unedited *orfB *transcripts may lead to impaired biogenesis and uncoupling or decreased phosphorylation activity of the F_1_F_0 _ATPase complex. In the present study, explanations for the absence of *orf*B transcript editing include, hydrophobicity alteration of the translated product; the lack of Phe58 in place of Leu in the absence of editing may adversely affect membrane attachment function; or the reduction of α-helix and extended coil in the protein may cause the malfunction of subunit 8 of the F_1_F_0_-ATPase complex.

## Conclusions

The study was initiated to elucidate the molecular genetic element(s) of the mt-genome in a CMS rice line with Wild Abortive (WA) cytoplasm that may be involved in causing male sterility. The study has clearly identified a putative CMS-associated mt-gene in the WA cytoplasm of rice. Studies are currently on-going to determine the functional role of the polymorphic *orfB *gene in causing cytoplasmic male sterility in the *APMS-6A *rice line (an *indica *cultivar with WA cytoplasm). Because hybrid seed production in rice is based primarily on the WA-type of CMS, these results may serve to develop future breeding strategies. The CMS system can be engineered to use a transgenic approach to more fully realize hybrid vigor in rice.

## Methods

### Plant materials

The plant materials utilized in this study included a three-line CMS system of *APMS-6A*, a WA type of CMS rice line (*Oryza sativa *subsp. *indica*); an isonuclear maintainer line with normal cytoplasm, *APMS-6B*; and a standard cytoplasm restorer line *BR-1870*. The lines were obtained from Acharya Ranga Agricultural University, Hyderabad, India. *APMS-6A *was derived from repeated backcrossing of *PR108*, an indigenous *indica *rice cultivar of Northern India with a CMS line, *IR58025A*. Additionally, the WA-CMS line *IR58025A*, its maintainer *IR58025B*; and non WA-CMS line *Kalinga 32A *and its maintainer *Kalinga 32B *(source: Central Rice Research Institute, Cuttack, India) were also utilized. The seeds of all lines were germinated in the dark at 37°C and grown under outdoor rice growth conditions to maturity.

### Primers

The primer sequences used in the study are provided in Table 1. The primers were synthesized in our laboratory using a DNA/RNA Synthesizer, Model 392 (Applied Biosystems).

**Table 1:** Description of primers used in the study

Primers	Base sequence 5' to 3'	Tm	Purpose
atp6-5'	GCTG**GGATCC**ATGAATTTCGATCACAATCATG	62°C	PCR primer for the *atp6 *gene probe

atp6-3'	CGGC**GAGCTC**TTACTCATTTTGATGGAGATT	62°C	PCR primer for the *atp6 *gene probe

atp9-5'	CGGC**GGATCC**ATGTTAGAAGAAGGAGCTAAATA	63°C	PCR primer for the *atp9 *gene probe

atp9-3'	CGGC**GAGCTC**CTATTTGCAAAGAGAGATATC	63°C	PCR primer for the *atp9 *gene probe

atpA-5'	ATAT**CTGCAG**CATGGAATTCTCACCCAGAGCTGG	66°C	PCR primer for the *atpA *gene probe

atpA-3'	AGCA**GGATCC**GAAGCGGTGGCTGCTACAA	66°C	PCR primer for the *atpA *gene probe

orfB-5'	GCTG**GGATCC**ATGCCTCAACTTGATAAATTGAC	63°C	PCR primer for the *orfB *gene probe T-PCR

orfB-3'	CGGC**GAGCTC**TTAGATTATGCTTCCTTGCC	64°C	PCR primer for the *orfB *gene probe T-PCR

AOT	GCGGCCAC**GGATCCGTCGAC** T_15_V(A/G/C)N(A/T/G/C)	-	Oligo-dT adaptor primer for 3' RACE of *orfB *gene

O-GSP1	CGAC**GGATCC**AAGAATTTGGAAGATATCTT	66°C	5' Gene-specific primer for 3' RACE of *orfB *gene

RACE AMP	GCGGCCAC**GGATCCGTCGAC**	62°C	3' RACE primer

Corf	GTCA**GGATCC**GGTGCTAAAACCTTTTCTC	62°C	3' Gene-specific primer for 5' RACE of *orfB *gene and RT-PCR

AAP	**GGCCACGCGTCGACTAGT**ACGGGIIGGGIIGGGIIG	-	Abridged anchor primer for 5' RACE of *orfB *gene

AUAP	**GGCCACGCGTCGACTAGT**AC	-	Abridged universal amplification primer for 5' RACE of *orfB *gene

orfB-UTR	TAGC**AAGCTT**CTACTACAGTATCGGCCTCG	66°C	3' Gene-specific primer for preparation of 1.1 kb specific northern probe.

Mtg-1	TCGA**GAGCTC**GGATAATCCGCATCAAGAAG	62.5°C	5' Gene-specific primer for preparation of 1.1 kb specific northern probe and RT-PCR.

FP-24	CGCCAGGGTTTTCCCAGTCACGAC	63°C	pUC18 forward sequencing primer

RP-24	AGCGGATAACAATTTCACACAGGA	54°C	pUC18 reverse sequencing primer

Restriction sites indicated in bold.

### Isolation of genomic DNA and RFLP analysis

Mitochondrial genomic DNA (mt-DNA) was isolated from mitochondrial fraction obtained from young rice leaves, following the CTAB method of [46]. Aliquots of DNA (10 μg) were digested with restriction enzymes *Bgl*II, *Sca*I, *Dra*I, *Eco*RI, and *Hind*III, as per the manufacturer's (Roche Molecular Biochemicals, Mannheim) instructions and fractionated on a 0.8% agarose gel together with *Eco*RI and *Hind*III digested phage λ DNA as the molecular weight marker.

The gene fragments used as probes were amplified from the restorer rice line by PCR using the appropriate primers (Table 1) in a thermocycler (GeneAmp System 9600, Perkin Elmer, USA) under the following reaction conditions: initial denaturation at 94°C for 4 min; followed by 30 cycles at 94°C for 30 s, 55°-60°C for 30 s and 72°C for 1 min; and a final extension for 7 min at 72°C. The annealing temperature was estimated according to the melting temperatures (T_m_) of the primer. The primers were designed on the basis of published sequences Acc #. X51422 [47] for the *atpA *gene; Acc #. X16936 [48] for the *atp9 *gene; Acc #. S59890 [28] for the *atp6 *gene; and Acc #. DQ167399 [49] for the *orfB *gene. PCR products were digested with restriction enzymes (sites of which were present as per design in the respective primers) followed by cloning with the pUC18 vector, transformed into *E. coli *DH10B cells and sequenced.

The gene probes (Table 2) were radiolabeled with [^32^P]dCTP (3500 Ci/mmol) by random priming using the Rediprime II DNA Labeling System (GE Healthcare, USA), following the manufacturer's instructions. Prehybridization and hybridization of Southern blots were performed in CHURCH buffer (0.25 M phosphate buffer; 1 mM EDTA; 7% sodium dodecyl sulphate (SDS), 1% BSA) at 65°C for 2 hr and 18 hr, respectively in a hybridization oven/shaker (GE Healthcare, USA). The blots were washed 3 × for 20 min in 2× sodium chloride and sodium citrate solution SSC, 0.1% SDS at 50°C; 0.5× SSC, 0.1% SDS at 55°C; 0.1× SSC, 0.1% SDS at 60°C. Autoradiographic exposure was carried out at -70°C.

**Table 2:** Probes used in the RFLP analysis to analyse different mitochondrially-encoded genes

Gene probe	Source	Fragment size	Description
*atp9*	Restorer rice line	263 bp	Complete coding region cloned as a *Bam*HI-*Sac*I fragment

*atpA*	Restorer rice line	720 bp	Partial coding region (lacking C-terminus) cloned as a *Pst*I-*Bam*HI fragment

*atp6*	Restorer rice line	876 bp 350 bp	Partial coding region (lacking C-terminus) cloned as a *Bam*HI-*Bgl*II fragment. Partial 3' UTR cloned as *Eco*RI-*Hin*dIII fragment.

*orfB*	Restorer rice line	468 bp	Complete coding region cloned as a *Bam*HI-*Sac*I fragment.

These gene probes were amplified from the restorer rice line by PCR, then cloned and sequenced.

### Isolation of mitochondrial RNA

Mitochondria were isolated from seven-day-old etiolated rice seedlings following the method of [50]. Mitochondrial RNA (mt-RNA) was isolated using a hot-phenol extraction method [51]. RNA quality was verified by agarose gel electrophoresis and the quantity estimated by UV- spectrophotometry. Aliquots of RNA (15 μg) were treated with 15 units of RNase-free DNase I (Roche Molecular Biochemicals, Mannheim) in the presence of MgCl_2 _(10 mM) at 28°C for 30 min.

### Northern blot analysis

Aliquots of mt-RNA (10 μg) were fractionated on a 1.2% agarose gel containing 6% formaldehyde (denaturing condition) adjacent to a RiboRuler™ High Range RNA Ladder (Fermentas, Canada). RNA was transferred to solid support (Hybond N^+^, GE Healthcare, USA) in 20× SSC by 3 hrs of vacuum transfer in a vacuum blotter (Model 785, Bio-Rad). The blots were hybridized with radiolabelled probes, as previously described for Southern blots. However, prehybridization, hybridization and washing were carried out at 42°C.

### Development of mitochondrial cDNA library

cDNA libraries were constructed from sterile and fertile rice lines using the Time Saver cDNA Synthesis Kit (GE Healthcare, USA). Double-stranded cDNA was first synthesized using random hexamer primers from 20 μg of DNase I-treated mt-RNA, following the manufacturer's instructions. Following second-strand cDNA synthesis and adaptor ligation, the adaptor-ligated cDNA was purified using a Sephacryl S-300 HR (GE Healthcare, USA) column. The purified cDNA was ligated to pUC18 DNA digested with *Eco*RI and treated with shrimp alkaline phosphatase (Fermentas, Canada); and subsequently transformed into chemically-competent *E. coli *DH10B cells. The libraries were amplified and screened according to [52]. DNA of the positive clones was sequenced using an automated ABI PRISM^® ^3100 Genetic Analyzer and BigDye^® ^Terminator v1.1 Cycle Sequencing Kit (Applied Biosystems, USA).

### RT-PCR

First-strand cDNA was synthesized from 3 μg of DNase I-treated mt-RNA using Superscript II reverse transcriptase (RT; Gibco BRL, USA) at 48°C for 60 min with the orfB-3' primer. The reaction was terminated by heating at 70°C for 15 min and then immediately chilled. The first-strand cDNA was subsequently treated with RNase H (Fermentas) for 1 hour at 37°C, according to the manufacturer's protocol. The *orfB *gene coding region was amplified by reverse transcription (RT) PCR with Deep Vent DNA polymerase (New England Biolabs, USA) and the orfB-5' and orfB-3' primers under the following reaction conditions: initial denaturation at 94°C for 4 min; followed by 30 cycles at 94°C for 30 s, 58°C for 30 s, 75°C for 1 min; and a final extension at 75°C for 7 min.

In addition, first strand cDNA was synthesized with the Corf primer using conditions similar to those already described. The partial *orfB *CDS (201 bp) including the entire 5'-UTR was amplified using Corf and Mtg-1 primers under the following reaction conditions: initial denaturation at 94°C for 4 min; followed by 30 cycles at 94°C for 30 s, 58°C for 30 s, 75°C for 1 min; and a final extension at 75°C for 7 min. The amplified products were cloned in the pUC18 vector and the positive clones were sequenced, as previously described.

### Poly (A) tailing and 3' rapid amplification of cDNA ends (RACE)

RACE was carried out using the 3' RACE System Kit (Gibco BRL, USA). Poly (A) tails were added to 5 μg of DNase I treated mt-RNA using poly(A) polymerase (Gibco BRL, USA) by incubation for 2 hr at 37°C according to the manufacturer's instructions. Polyadenylated mt-RNA was purified by phenol-CHCl_3 _extraction and subsequent overnight ethanol precipitation. First-strand cDNA was synthesized using an oligo(dT) adaptor primer (AOT; Table 1) at 48°C for 60 min with Superscript II RT (Gibco-BRL). PCR was carried out with polyadenylated cDNA as the template, using (i) the gene-specific primer O-GSP1 (designed on the basis of cDNA library clone sequence analysis), and (ii) a RACE-AMP primer provided in the kit that anneals to the poly(A) tail and Deep Vent DNA polymerase (New England Biolabs, USA). The reaction conditions were as follows: initial denaturation at 94°C for 4 min; 30 cycles of 94°C for 30 s, 60°C for 30 s, 75°C for 1 min; and a final extension at 75°C for 7 min. The products were digested with restriction enzymes and cloned at compatible sites in the pUC18 vector according to [53] and sequenced.

### 5' RACE

5' RACE was carried out on DNase I treated mt-RNA using the 5' RACE system, version 2.0 (Gibco BRL), following the manufacturer's protocol. First-strand cDNA was synthesized from 3 μg of mt-RNA using the Corf primer (Table 1) at 42°C for 1 h. The sample was then treated with 1 μl of RNase H for 1 h at 37°C. The resulting cDNA was purified by passing it through a GlassMAX spin cartridge (Gibco BRL) with a cut-off value of 200 bp and eluted with 50 μl of double-distilled water (as per the manufacturer's instructions). The purified cDNA was then subjected to 5' tailing with 200 μM dCTP using terminal deoxynucleotidyl transferase (Fermentas) for 15 min at 37°C. The first round of PCR was performed with 2.5 μl of 'C'-tailed cDNA as the template, Deep Vent DNA polymerase (New England Biolabs), the 5' RACE abridged primer (AAP) provided in the kit and the gene-specific Corf primer. Reaction conditions were as follows: initial denaturation at 94°C for 4 min; followed by 30 cycles at 94°C for 30 s, 56°C for 30 s, 75°C for 1 min; and a final extension at 75°C for 7 min. Subsequently, the second round of PCR was conducted with 1 μl of the first round product as template, using the abridged universal amplification primer (AUAP) provided in the kit and the Corf primer. The cycling conditions were as described above. The 5' RACE products were cloned and the positive clones were sequenced.

### Aceto-carmine stain test for pollen fertility

Fertile pollen was differentiated from sterile pollen by nuclear staining (1% aceto-carmine) followed by viewing under a compound light microscope. Viable/fertile pollen exhibited high stainability (a dark pinkish color), whereas the nonfunctional/sterile pollen remained unstained (or a faint pinkinsh color). Aceto-carmine was prepared by dissolving 1 gm of carmine (EMerck, Darmstadt) in boiling 45% glacial acetic acid followed by filtering.

## Authors' contributions

The studies were conceived and planned by SKS. All experimental works were carried out at different stages of the study by SD, AC, SS and PC. Time to time technical guidance, whenever necessary, was offered by SKS, DB, MKM and AB. The manuscript was edited and prepared by SKS along with SD and AC. All authors read and approved the final manuscript prepared for submission by SKS.

## Supplementary Material

Additional file 1 **Size of hybridized DNA fragments in kb**. Sizes of the DNA restriction fragments obtained from Southern hybridization. Click here for file

Additional file 2 **Size of hybridized DNA fragments in kb**. Sizes of DNA fragments hybridized to probe in kb (RFLP with 468 bp *orfB *CDS probe) Click here for file

Additional file 3 **Chi square test**. Chi square test for goodness of fit of inheritance of trihybrid pattern of epistatic gene interaction. Click here for file

Additional file 4 **Gene displaying expression profiles**. Multiple sequence alignment of the *atp8 *gene from various monocot and dicot plants with the *orfB *CDS of WA sterile rice. (A) Sorghum (Accession No. DQ984518), wheat (Accession No. AP008982) and *Oryza *(Accession No. BA000029) with the *orfB *CDS of WA sterile rice and (B) *Beta vulgaris *(Accession No. NC002511) and *Daucus carota *(Accession No. AY007818) with the *orfB *CDS of WA sterile rice. The alignment was performed with Jellyfish version 1.3 software provided by biowire.com. Click here for file
