# Supplementary material for: An unedited 1.1 kb mitochondrial orfB gene transcript in the Wild Abortive Cytoplasmic Male Sterility (WA-CMS) system of Oryza sativa L. subsp. indica
Source: BMC Plant Biol. 2010 Mar 2;10:39. doi: 10.1186/1471-2229-10-39 (PMC2848759; doi:10.1186/1471-2229-10-39)
Supplement: Additional file 1 — Size of hybridized DNA fragments in kb. Sizes of the DNA restriction fragments obtained from Southern hybridization. [file 1471-2229-10-39-S1.DOC]

#### Additional File 1

### Sizes of the DNA restriction fragments obtained from Southern hybridization with the different probes

| Probe used | Sizes of fragments hybridized to probe in kb | | | | | Rice Lines |
| --- | --- | --- | --- | --- | --- | --- |
|  | *Bgl*II | *Sca*I | DraI | EcoRI | HindIII |  |
| 876 bp partial coding region of *atp6* gene | 1.4 | 12.0 | 21.3 | 5.2 | 20.8 | APMS-6A |
|  | 1.4  3.4 | 1.2  12.0 | 13.0  24.0 | 2.5  21.0 | 18.0  20.0 | APMS-6B |
|  | 1.4  3.4 | 1.2  12.0 | 13.0  24.0 | 2.5  21.0 | 18.0  20.0 | *BR-1870* |
| 3’ UTR of *atp6* gene | 3.3 | 1.15 | 21.3 | 5.2 | 20.8 | APMS-6A |
|  | 3.1 | 1.15 | 13.0  24.0 | 2.5  21.0 | 18.0  20.0 | APMS-6B |
|  | 3.1 | 1.15 | 13.0  24.0 | 2.5  21.0 | 18.0  20.0 | BR-1870 |
| Coding region of *atp9* gene | 21.1 | 12.5 | 19.2 | 18.5 | 1.45  1.70 | APMS-6A |
|  | 21.1 | 12.5 | 19.2 | 18.5 | 1.45  1.70 | APMS-6B |
|  | 21.1 | 12.5 | 19.2 | 18.5 | 1.45  1.70 | BR-1870 |
| 720 bp partial coding region of *atpA* gene | 12.0  2.1 | 3.0 | 1.5 | 2.5 | 4.2 | *APMS-6A* |
|  | 12.0  2.1 | 3.0 | 1.5 | 2.5 | 4.2 | APMS-6B |
|  | 12.0  2.1 | 3.0 | 1.5 | 2.5 | 4.2 | BR-1870 |
