# Supplementary material for: An unedited 1.1 kb mitochondrial orfB gene transcript in the Wild Abortive Cytoplasmic Male Sterility (WA-CMS) system of Oryza sativa L. subsp. indica
Source: BMC Plant Biol. 2010 Mar 2;10:39. doi: 10.1186/1471-2229-10-39 (PMC2848759; doi:10.1186/1471-2229-10-39)
Supplement: Additional file 2 — Size of hybridized DNA fragments in kb. Sizes of DNA fragments hybridized to probe in kb (RFLP with 468 bp orfB CDS probe) [file 1471-2229-10-39-S2.DOC]

**Additional File 2**

RFLP with 468 bp *orfB* CDS probe

| Sizes of DNA fragments hybridized to probe in kb | | | | | **Rice Lines** |
| --- | --- | --- | --- | --- | --- |
| *Bgl*II | *Sca*I | *Dra*I | *Eco*RI | *Hin*dIII |
| 5.1 | 3.0 | 4.1 | 2.4  3.4 | 1.3 | *APMS-6A* |
| 4.2 | 6.2 | 3.8 | 4.0  3.4 | 12.5 | *APMS-6B* |
| 4.2 | 6.2 | 3.8 | 4.0  3.4 | 12.5 | *BR-1870* |
