# Supplementary material for: An unedited 1.1 kb mitochondrial orfB gene transcript in the Wild Abortive Cytoplasmic Male Sterility (WA-CMS) system of Oryza sativa L. subsp. indica
Source: BMC Plant Biol. 2010 Mar 2;10:39. doi: 10.1186/1471-2229-10-39 (PMC2848759; doi:10.1186/1471-2229-10-39)
Supplement: Additional file 3 — Chi square test. Chi square test for goodness of fit of inheritance of trihybrid pattern of epistatic gene interaction. [file 1471-2229-10-39-S3.DOC]

**Additional File 3**

Chi-Square Test

| Phenotypes | Observed # (o) | Expected # (e) | d (o-e) | d^2^ | d^2^/e |
| --- | --- | --- | --- | --- | --- |
| 2008 Crop:  Fertile Pollen | 160 | 159.4 | 0.6 | 0.36 | 0.002 |
| Sterile Pollen | 2 | 2.53 | - 0.53 | 0.28 | 0.11 |
| x^2^= 0.112; df=1 | | | | | **0.112** |
| 2009 Crop:  Fertile Pollen | 209 | 208.68 | 0.32 | 0.102 | 0.0005 |
| Sterile Pollen | 3 | 3.31 | - 0.31 | 0.096 | 0.029 |
| x^2^= 0.0295; df=1 | | | | | **0.0295** |
