# Supplementary figures and images for: An unedited 1.1 kb mitochondrial orfB gene transcript in the Wild Abortive Cytoplasmic Male Sterility (WA-CMS) system of Oryza sativa L. subsp. indica
Source: BMC Plant Biol. 2010 Mar 2;10:39. doi: 10.1186/1471-2229-10-39 (PMC2848759; doi:10.1186/1471-2229-10-39)

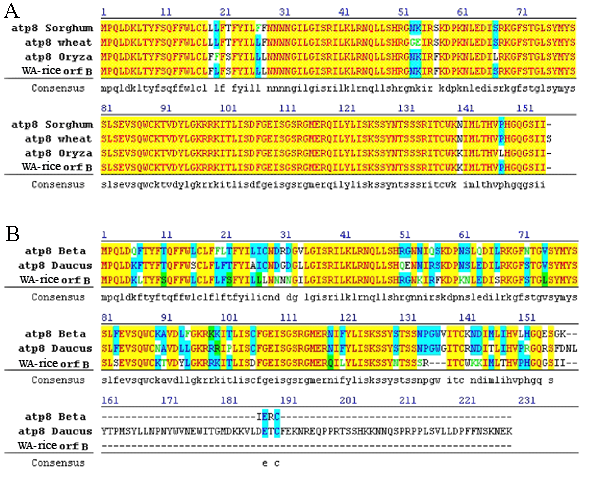

Supplement: Additional file 4 — Gene displaying expression profiles. Multiple sequence alignment of the atp8 gene from various monocot and dicot plants with the orfB CDS of WA sterile rice. (A) Sorghum (Accession No. DQ984518), wheat (Accession No. AP008982) and Oryza (Accession No. BA000029) with the orfB CDS of WA sterile rice and (B) Beta vulgaris (Accession No. NC002511) and Daucus carota (Accession No. AY007818) with the orfB CDS of WA sterile rice. The alignment was performed with Jellyfish version 1.3 software provided by biowire.com. [file 1471-2229-10-39-S4.TIFF]
